# The relationship between forest fire and deforestation in the southeast Atlantic rainforest

**DOI:** 10.1371/journal.pone.0286754

**Published:** 2023-06-02

**Authors:** Cheila Flávia de Praga Baião, Fabrícia Cristina Santos, Marcos Paulo Ferreira, Rafael Beltrame Bignotto, Rafael Felipe Guatura da Silva, Klécia Gili Massi

**Affiliations:** 1 Programa de Pós Graduação em Desastres Naturais, Universidade Estadual Paulista "Júlio de Mesquita Filho" (Unesp)/ Centro Nacional de Monitoramento e Alertas de Desastres Naturais (Cemaden), São José dos Campos, São Paulo, Brazil; 2 Instituto de Pesquisas Ambientais (IPA)/ Secretaria de Infraestrutura e Meio Ambiente (SIMA), Viveiro Florestal de Taubaté, Taubaté, São Paulo, Brazil; 3 Departamento de Engenharia Ambiental, Instituto de Ciência e Tecnologia, Universidade Estadual Paulista "Júlio de Mesquita Filho" (Unesp), São José dos Campos, São Paulo, Brazil; Van Lang University: Truong Dai hoc Van Lang, VIET NAM

## Abstract

Given the scarcity of studies relating fire to deforestation in the Atlantic Forest and great economic and ecological importances of this biome, this work aimed to investigate this relationship in the Atlantic Forest of the State of São Paulo, trying to answer whether deforestation is related to fire events in up to three years, if there are regions most affected by this relationship and what land use and land cover predominates after fire and deforestation in these areas. The study was carried out in Evergreen Forest and Semideciduous Seasonal, along the time series from 2000 to 2019 using the MapBiomas Project database to survey deforested and burned sites with moderate to high severity fires. Burning positively influenced deforestation in EGF in eight of 19 years studied (2001, 2004, 2005, 2007, 2008 and 2009, 2013 and 2015), while only for three years in the SSF. Burning followed by deforestation corresponded to only 3.2% of the total deforestation, located mainly in the eastern region of the state with the highest density in the EGF. Most of these areas have been converted to agriculture. This study provides the first indication that, generally, fire is not a driver of deforestation in the southeast Atlantic Forest.

## Introduction

The Atlantic Forest biome is a biodiversity hotspot [1]. Due to intense deforestation and human disturbance that mostly occurred in the first half of the 19th century [2], only about 13% of Atlantic Forest biome native vegetation cover remains in Brazil [3, 4]. Nature reserves protect only 9% of the remaining forest [5]. This percentage of native cover has been maintained, but there is a concern that the ongoing loss of older native forests, mostly on flatter terrains, have been hidden by the increasing gain of younger native forest cover, mostly on lands not suited for mechanized agriculture [6]. The MapBiomas Project showed that, from 1985 to 2021, 8.3 million hectares of forest cover were converted to large scale agriculture lands in the Atlantic Forest Biome [7] and this loss of older native forest might be associated with forest fires.

Every year, wildfires burn more than 400 million hectares worldwide [8] and shape the structure and diversity of all biomes [9]. Recently, some portions of the Atlantic Forest biome have also been experiencing an increase in area burned by wildfires [10]. Humans are a major force driving many fire regimes around the globe [11], and, in Brazil, fire is often a key process when considering the drivers of forest loss [12] like those that happened in the Amazon region during the summer of 2019, related to commodity-driven deforestation or agriculture [13]. In the Atlantic Forest, fire may also be human-driven, especially for agriculture practices.

Fires in tropical forests, which are not natural events, burn surface fuel as dry leaves and grasses, generally cause extensive top-kill in small trees [14] and leaf-fall in larger trees (due to heating), allowing increased light to the forest floor and a establishment of invasive grasses, changing likelihood of recurrent fires [15]. When subjected to such conditions, non-adapted species can be selectively excluded from the community, generating a drastic change in species composition, biomass and vegetation cover [16–18]. Depending on severity, surface fire can lead to small changes in cover and mortality of the structurally dominant vegetation (low severity) to large vegetation mortality (high severity) [19–21].

Despite the importance of the Atlantic rainforest and fire threat, studies on impacts of disturbances such as burnings over this biome are scarce [17, 18–24]. In Brazil, burning is forbidden as a management tool (unless under environmental permission or when done by traditional communities: [25, 26]. In rural sites, fires are used to renew pasture and agriculture sites and to clean trash and these fires may go uncontrolled to near forests. In addition, they can be intentionally used to degrade forests and regenerating sites and promote deforestation, especially for commodities agriculture and urbanization purposes. Fire-deforestation relations, well understood in Amazon, might happen differently in the Atlantic Forest, as this biome is fragmented and inserted in an anthropogenic landscape.

Therefore, the objective of our study is to contribute to filling this knowledge gap by investigating how deforestation dynamics in the Atlantic Forest Biome are related to moderate to high severity fire events, in patches of evergreen and seasonal forest in southeast Brazil, which are fire-sensitive ecosystems. Specifically, we aimed to answer (i) Did deforestation happen after burnings (one, two and three years later) in sites of the Atlantic rainforest biome? (ii) Are some regions most affected by deforestation related to fire than others? And (iii) What was the predominant land cover after fire and deforestation? We expected fire as a driver of deforestation in the Brazilian Atlantic Forest and commodities agriculture as a driver of both impacts.

## Material and methods

### Study area

Sao Paulo state is located in southeast Brazil (25º21”29’S and 44º9”41’W) and contains the largest industrial park in the country (responsible for 32.2% of the Brazilian GDP: [27]) and one of the richest economies in the world, which began in the transition between the 18th and 19th centuries with coffee plantations, was substituted later for sugarcane and currently holds an industrial, urban and cultural protagonism [28]. The state is divided into 645 municipalities over an area of 248,219.94 km^2^ (2.9% of the Brazilian territory) and a population of almost 45 million inhabitants (about 21% of its entire population), being the most populous in the country [29]. Native vegetation of Sao Paulo is distributed among Atlantic Forest and Cerrado Biomes, with original proportional area of 67.3% and 32.7%, respectively; currently only 22.9% of the state area is covered with both Biomes, where the largest portion of Atlantic Forest has 10.1% are Ombrophilous Dense or evergreen forest (EGF), and 7% are seasonal semideciduous forests (SSF) [28]. EGF is occupied predominantly by perennial trees, epiphytes, with a uniform canopy and not subjected to dry periods, while SSF occurs under a climate with a well-defined dry season, leading to 20–50% loss of leaves in tree canopy [30]. EGF has 76368 patches averaging 31.57 ha (±1,902.67), while SSF has 201,560 patches, with smaller sizes, averaging 9.16 ha (±79.86), but more scattered along the state [28].

### Dataset

Boundaries of Evergreen Forest (EGF) and Seasonal Semideciduous Forest (SSF) were obtained from vector files of São Paulo Forest Inventory [28] from DataGeo System, infrastructure of environmental spatial data of the State of São Paulo (https://datageo.ambiente.sp.gov.br/).

The Burned and deforested areas of the study area were obtained from two datasets of MapBiomas platform, a multidisciplinary network that uses cloud processing and methodologies of pattern recognition, to generate historical series of annual maps of land use and land cover and fire scars, from 1985 to 2020, from images from sensors on board Landsat satellites, with a resolution of 30 m (https://mapbiomas.org/). Although Sentinel satellite images offer images of 10 m of spatial resolution, which would increase the accuracy of this work, its data only started in 2014, not fitting the study design. In addition, Mapbiomas fire and deforestation data have been used in several scientific studies [6, 7, 31–33]. The first dataset was annual fire scars data, evaluated from 2000 to 2016, that can detect fires with a dNBR (Normalized Burn Ratio Difference) pre-post fire greater than 0.25, (moderate to high severity fire [20, 21]) and has an accuracy of 97% [31],. And the second dataset was deforestation and regeneration data, with overall accuracy in Atlantic Forest of 87,3% [7] were evaluated from 2000 to 2019 (last year of this collection). The last year of fire scars was 2016 in order to overlap fire data with deforestation in the current and following three years. The set of deforestation and regeneration provides the annual land use and land cover classes plus classes that indicate regeneration or deforestation in primary and secondary forests when they occur. If a certain site was deforested and it has not regenerated or converted into another land use and cover, it would be classified as deforested in different years; however, we believe this represents a very small percentage of the total deforestation, if it occurs, as rural land in São Paulo state is quickly destined for other uses after deforestation. Despite the fire dataset is available monthly, the deforestation dataset has no available monthly data, so the relationship between fire and deforestation was carried out annually. Matrix files were processed in Quantum Gis 3.16.11 software (https://www.qgis.org/en/site/).

### Data analysis

**Fig 1:**
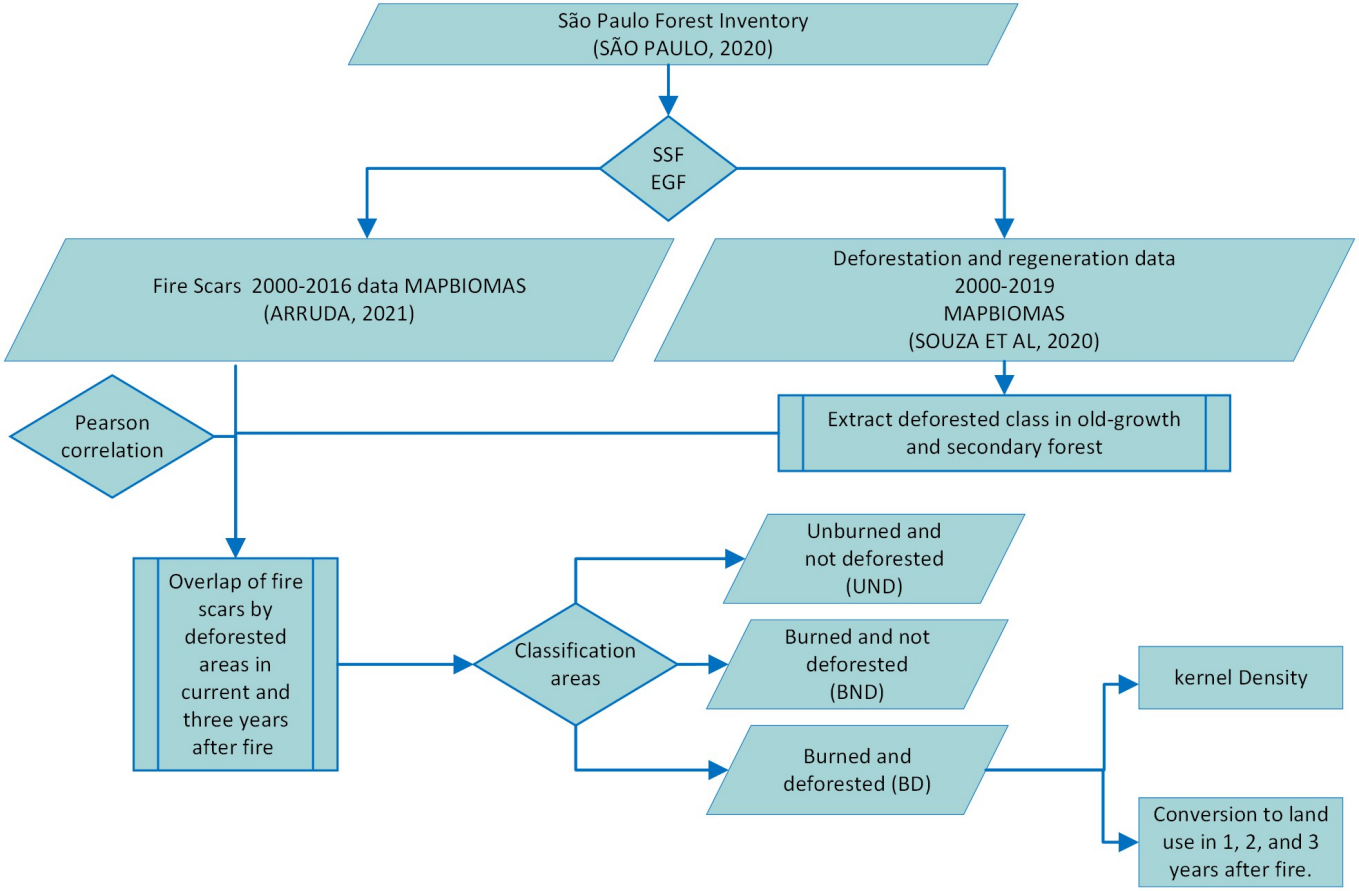
Methodological procedure for collecting and analyzing fire and deforested data.

Using Quantum Gis, area (size) and number of patches were calculated for burned and deforested areas separately in each year of the study period. To identify intersection between burned and deforested areas and land use and land cover conversion following fire-caused deforestation, fire files were overlapped with deforestation files in the current years and following three years thereafter (Fig 1). Thus, sites were classified in burned and not deforested (BND), burned and deforested (BD), when there was intersection between burned and deforested areas in current and three years later, and unburned and deforested (UBD). Area and number of intersections were calculated as well as conversions in each land use and land cover identified in the overlay. Conversions after deforestation were counted from 2001 onwards, since datasets were annual, and the time series of the study started in 2000.

We also performed a preliminary analysis to evaluate effects of distance from anthropogenic land cover (urban and agriculture) and of distance from watercourses on fires, but we did not identify significant correlation, as expected due to the fragmented and anthropogenic landscape nature of the Atlantic Forest in São Paulo state (data not shown).

To identify regions with greater occurrence of burning followed by deforestation, central points of the polygons of intersection were created to perform a heat map through the Kernel density. The mean of averages ($\overset{-}{x}$) added or subtracted from the mean of standard deviations (*σ*) were used to evaluate the best radius size. The kernel density function used was the quartic that weighs closer points more heavily than distant points, but decrease is gradual.

BD areas were then again overlapped with deforestation and regeneration maps to identify land use and land cover (LULC) in the three years following fire and deforestation (Fig 1).

Pearson correlation between extension of burned areas (explanatory variable) and deforested areas (response variable) of current year and until three years after fire was performed in order to assess whether the increase of burned areas also increased deforested areas, with Python programming language (version 3.6), which allows an interactive computing environment for editing and executing data analysis [34].

## Results

**Fig 2:**
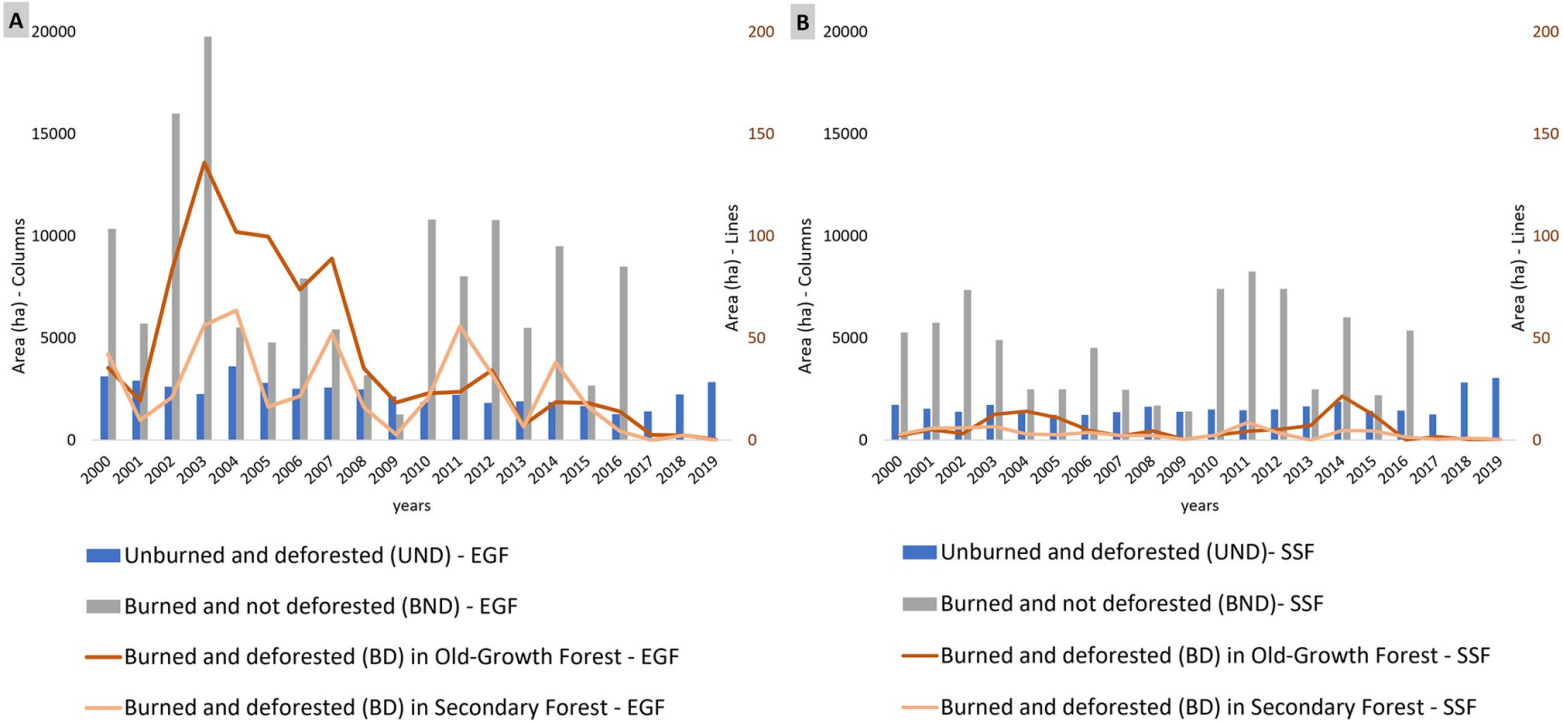
Area extension of unburned and deforested (UNB), burned and not deforested (BND) and burned and deforested (BD). Frequency of these areas in São Paulo State in the period 2000 to 2019 for Evergreen Forest (A) and Semideciduous Forest (B).

In Evergreen Forest (EGF), deforestation represented 16,026 patches in a total area of 47,560.14 ha, being 55.2% of old-growth forest and 44.8% of secondary forest (Table 1 and Fig 2A). Most deforested forest patches (96.3%) had between 2 and 5 ha (old-growth: 3.05 ± 1.74 ha and secondary: 2.86 ± 1.29 ha). Although the largest number of deforested areas were found in 2004, there was a decrease trend in deforested areas between 2000 and 2016, rising again between 2017 and 2019 (Fig 2A). Fire scars in EGF were verified in 32,846 patches in a total area of almost triple of deforestation. Most of burned forest patches (95.9%) were between 2 and 10 ha (4.25 ± 3.7 ha). Burned areas in EGF showed an increasing trend between 2000 and 2003 (with the highest number in 2003), with a decrease between 2004 and 2009, and increasing again in 2010 (Fig 2A).

**Table 1:** Deforestation (2000–2019), fire scar (2000–2016) and fire-related deforestation in Evergreen Forest (EGF) and Semideciduous Seasonal Forest (SSF) in São Paulo state in current year and three years following fire.

	Forest	Nº patches	Area (ha)	Old-growth(%)	Secondary (%)	Average (ha)
**Deforestation**	EGF	16,026	47,560.14	55.2	44.8	2.96 (±1.55)
	SSF	40,469	32,792.12	32.3	67.6	0.81 (±0.85)
**Fire scar**	EGF	32,846	139,545.60	-	-	4.25 (±3.7)
	SSF	19,454	77,699.39	-	-	3.99 (±3.42)
**Intersection**	EGF	952	1,316.49	63.6	36.4	1.38 (±1.5)
	SSF	399	179.9	64.6	35.4	0.45 (±0.78)
**Fire-deforestation (%)**	EGF		2.8%			
	SSF		0.5%			

Burning and deforestation in EGF occurred in 952 patches, with a total area of 1,316.49 ha, of which 63.6% was old-growth (1.46 ± 1.56 ha) and 36.3%, secondary (1.26 ± 1.36 ha). Most of BD patches had between 1 and 5 ha (51.68%), followed by (47.5%) patch size smaller than 1 ha. Deforestation and burning mainly occurred between 2002 and 2007 in old-growth forests, with a peak in 2003 (136 ha); in the secondary forest, 2004, 2007, 2011 and 2014 were peaks (Fig 2A).

For seasonal semideciduous forest (SSF), deforestation represented much more patches but with a minor area (≅33,000 ha), being 32.3% of old-growth and 67.6% of secondary forest (Table 1 and Fig 2B). Most of deforested forest patches (80.1%) were smaller than 1 ha and 19,4% were between 1 and 5 ha (old-growth: 0.95 ± 1.27 ha and secondary: 0.75 ± 0.62 ha). These areas remained with small fluctuations between 2000 and 2017, increasing in 2018 and 2019. The number of patches and area in Fire scars in SSF decreased by approximately half of EGF (Fig 2B).

Deforestation and burning in SSF represents 0,5% of total deforestation in this phytophysiognomy, of which 64.6% was old-growth (0.52 ± 0.57 ha) and 35.4%, secondary (0.36 ± 0.31 ha). Most of BD patches had 6 ha (91%). Deforestation and burning mainly occurred between 2003 and 2005 and between 2014 and 2015 in old-growth forest, while it occurred between 2001 and 2003 and in 2011 in secondary forest (Fig 2B).

**Fig 3:**
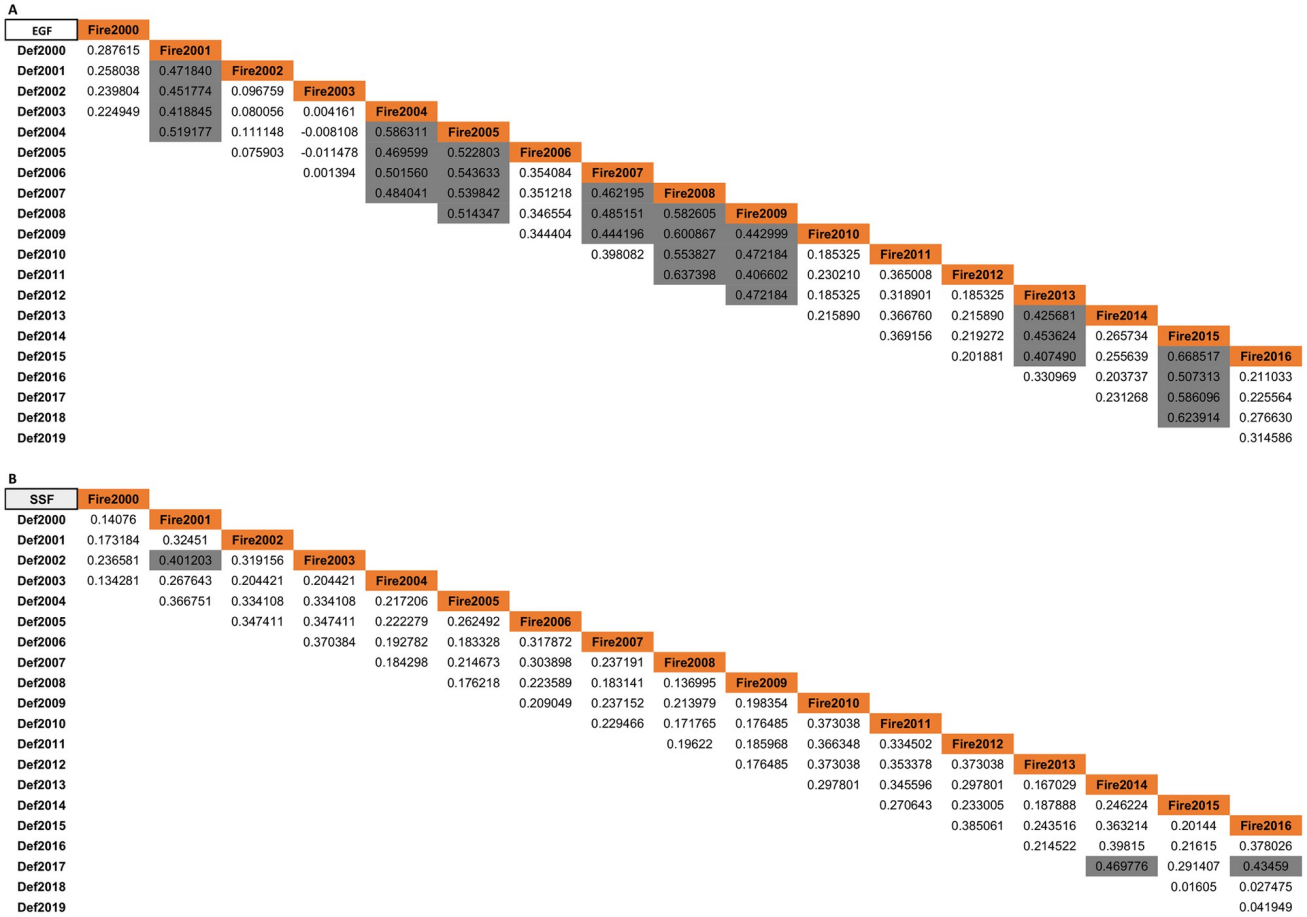
Pearson’s correlation between fire scar and deforestation areas. In current year within three years period in Evergreen Forest (EGF—A) and Seasonal Semideciduous Forest (SSF—B) (gray values represent p-value <0.0001).

We found significant correlation between BD areas for EGF in about half of studied years (Fig 3A). In 2001, 2004, 2005, 2007, 2008 and 2009, 2013 and 2015, fire was positively associated with deforestation in EGF, while in 2001, 2014 and 2016 fire influenced deforestation in SSF (Fig 3). The R^2^ varied from 16% to 44% with an average of 26% in these years. Most of SSF correlations between burned areas and deforestation were not significant (Fig 3B).

Burnt and deforested areas were identified by Kernel density (radius of 74,284 m for EGF and 111,934 m for SSF ($\overset{-}{x}-\sigma$)) and occurred more intensely in northeast São Paulo state; while in EGF were especially in east to south sides, in Paraíba do Sul, Tietê, Piracicaba and Ribeira Valley water basins, in SSF, located in central portion of the state mainly in Perdões and Mogi-Guaçu Region.

**Fig 4:**
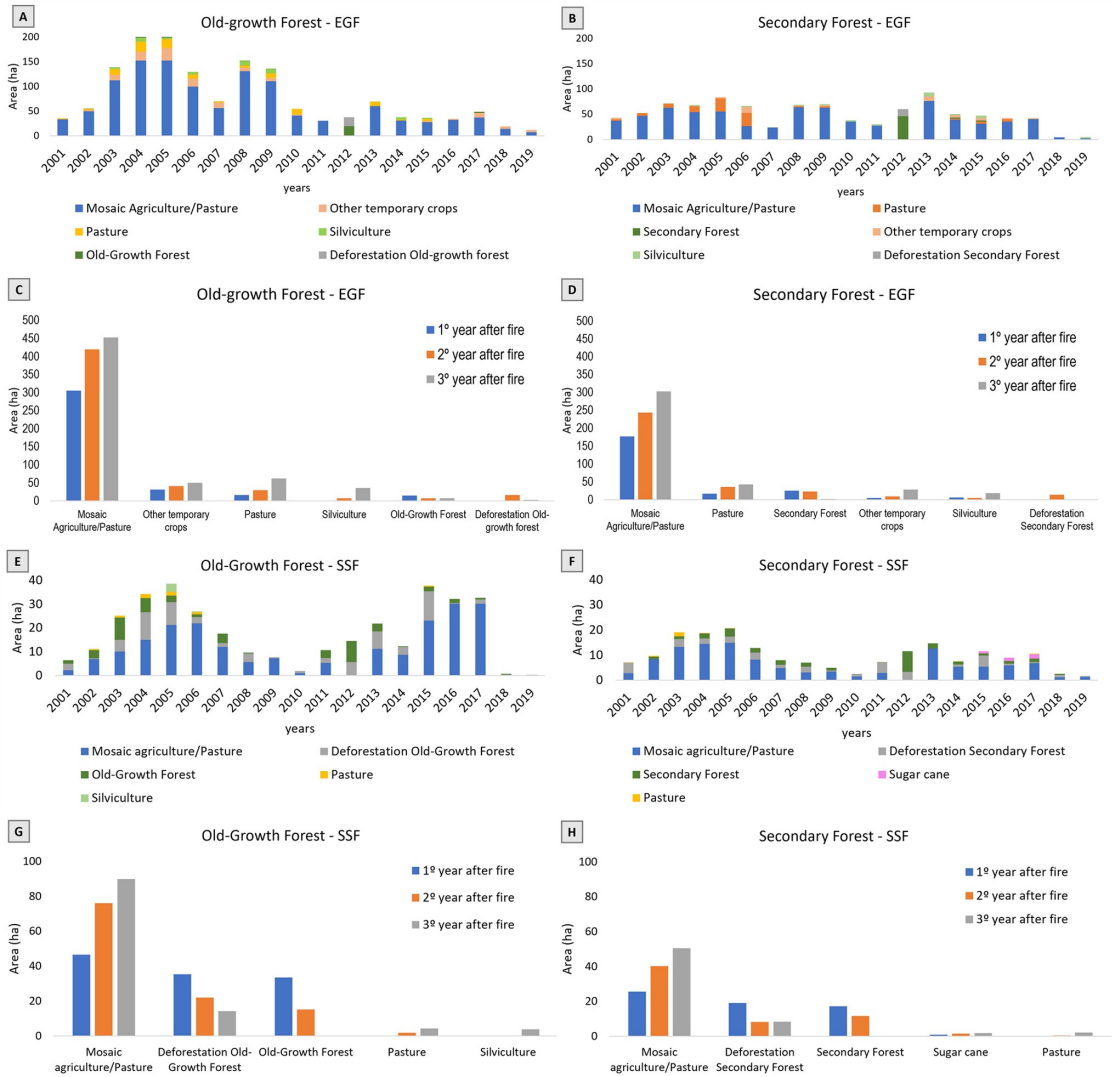
Conversion of LULC post-fire and deforestation. From 2001 and 2019 (Old-Growth Evergreen Forest—A; Secondary Evergreen Forest—B; Old-Growth Semideciduous Forest—E; and Secondary Semideciduous Forest—F) and first, second and third year after fire (Old-Growth Evergreen Forest—C; Secondary Evergreen Forest—D; Old-Growth Semideciduous Forest—G; and Secondary Semideciduous Forest—H). Only classes that exceed 1% of the total converted area are represented.

Conversion of deforested and burned forest patches were mainly to agriculture and pasture, both in EGF and SSF, for old-growth and secondary forest (Fig 4). Agriculture and pasture mosaic represented most of conversion for old-growth EGF forest (1,524.23 ha: 77.24%), followed by temporary crops, pasture and silviculture with 8.05%, 7.06% and 2.85%, respectively (Fig 4A). For EGF secondary forest (966.55 ha), agriculture and pasture mosaic represented 74.91% and pasture, 9.89%, other temporary crops, 4.44%, and silviculture, 3.14%. Only 5.24% of areas returned to secondary forest (Fig 4B). Agriculture and pasture mosaic represented most of conversion for old-growth SSF forest (348.80 ha: 60.84%), followed by pasture (1.74%), silviculture (1.08%) (Fig 4E). For SSF secondary forest (190.89 ha), agriculture and pasture mosaic represented 60.78%, sugar cane, 2.01%, and 1.24% for pasture (Fig 4F). Only 15.03% of areas returned to secondary forest.

Likewise, agriculture and pasture mosaic were predominant in the years following fire, increasing area from the first to third year. This pattern was the same both in old-growth and secondary forests of EGF and SSF (Fig 4C, 4D, 4G and 4H).

## Discussion

Analyzing number of forest patches, we found most of evergreen forest patches having fire scars and most of seasonal forest patches losing vegetation, while when we evaluated total area affected by fire, both evergreen and seasonal forests were quite significantly affected by burnings. The use of fire in rural lands is forbidden in Brazil, but fires are commonlly used to renew pasture and agriculture sites and to clean trash and these fires may go uncontrolled to near forests. In addition, burnings can be intentionally used to degrade forests and regenerating sites and promote deforestation, for agriculture and urbanization purposes.

Using fire in São Paulo state to manage agriculture, mainly related to sugarcane plantations [35], i.e., clean plantations, reducing aboveground biomass (especially leaves), and facilitate sugar cane harvest was a common practice in São Paulo state until 2002, when a state law regulated gradual elimination of fire for that purpose (Law n 11.241/2002: [36]). According to this regulation from 2011 to 2016, 50% to 80% of elimination of fire use in sugarcane mechanized sites should be pursued (in 2021 that would be 100%). Pastures also are cleaned and renewed through fire [37, 38], under environmental permission or illegally. In São Paulo state, most of land use and land cover and commodities agriculture is attributed to annual crops (mainly sugarcane) and pasture [39]. These agriculture and pasture areas border small (as we observed, most patches do not exceed 10 ha) and fragmented forest patches of the Atlantic Forest [5, 40], making them vegetation islands highly exposed to burnings [22], which explain our results.

Along the years there was a decreasing trend in deforested areas between 2003 and 2016 in EGF and a stationary deforestation pattern in SSF, both having raised again between 2017 and 2019. Our results indicate that command and control policies might have diminished deforestation rates in the Atlantic Forest in São Paulo state, especially after specific legislations regulating conservation and preservation of the biome (as the Atlantic Forest Law from 2006: [41]). Lately, deforestation rates and human pressure on biodiversity and ecosystems have been increasing, inflated by the recent dismantling of legal environmental framework by the Bolsonaro administration [42]. When we looked to overlapped BD sites, we found that they represented a minor proportion of forest patches and area, contrary to our expectations and to the processes of forest loss linked to burnings in other Brazilian regions [13, 43]. Also, the significant correlations between burned and deforested areas for EGF were small (eight of eighteen studied years), and almost none for SSF (three).

Thus, we may discuss that forest patches in São Paulo state are being burned and degraded, as our previous results showed, but they are not being cut down, probably because these remnants constitute legal reserves or permanent preservation areas, which are mandatory native vegetation protected areas inside rural land (according to New Forest Code, Law n 12.651/2012 [25]). Despite that is a positive result, degradation of forests by fire is a concerning issue that starts to be a conservation and restoration agenda of non-governmental organizations in Brazil and in São Paulo state, as TNC—The Natural Conservancy (personal communication). Historically, the Atlantic rainforest was deforested using fire [2], for large-scale agricultural purposes (sugarcane and coffee monocultures) leading to a rapid expansion of the human frontier.

Additionally, we found that regions most affected by deforestation related to fire in Atlantic Forest sites were evergreen forests and located in three important socioeconomic areas (Ribeira, Tietê/Piracicaba and Paraíba do Sul River basins). Firstly, the Ribeira Valley is a region of great scenic beauty and one of the best-preserved places in the Atlantic Forest, which, however, has been suffering from burnings [44]. It is a low human population density region with low-intensity agriculture as the main economic activity [45], but is target of a Strategic Development Plan in the next few years [46], which may alter native vegetation cover. In this region, fire might be used to degraded old-groth forests and facilitate environmental licensing, as in other regions. The Tietê and Piracicaba River basins are densely populated and urbanized regions, with land use dominated by large-scale and commodities agriculture [47]. Lastly, Paraíba Valley, with a growing trend of fires [10] aggravated by high pasture cover [32], which may be particularly threatening many important local restoration and conservation initiatives such as the Atlantic Forest Connection Project—GEF (Global Environmental Facility: https://conexaomataatlantica.mctic.gov.br/cma/portal/). Also, in these regions are located some fundamental protected areas, such as Serra do Mar state park, the largest conservation area of Atlantic Forest in Brazil (https://www.infraestruturameioambiente.sp.gov.br/pesm/), that fire occurrence may endanger. All these burned evergreen forests might be having biodiversity loss and changes in forest structure, functioning and ecosystem services [17, 18, 24].

Fire and deforestation of seasonal forests are happening in ecotonal regions with the Cerrado biome (a neotropical savanna), constituted of fire-influenced ecosystems [48], and more resilient to burnings. Despite that, fire is considered a threat even to Cerrado remnants of São Paulo state [49], if they did not follow their natural regimes. Then fire policies and management should be well planned and implemented in the two biomes that happen in São Paulo state.

Brazil’s Atlantic Forest is still losing native forests [6], mostly by the expansion of agricultural frontiers [13]. Our data corroborate this and show fire and forest deforestation followed by agriculture and pasture mosaic, as we expected, mainly in the third year after fire which differs from Amazon region, when conversion occurs immediately after fire and deforestation [50, 51]. Despite recent data of the Forest Inventory indicating vegetation cover increases in the São Paulo state [28], deforestation is still happening, driven by large-scale and commodities agriculture, such as sugarcane, cattle (for meat), soybean, orange and chicken (for meat) (http://www.iea.agricultura.sp.gov.br/out/index.php). The Atlantic Forest Law (Lei da Mata Atlântica, Law no 11.428/2006 [41]) prohibits deforestation of late secondary and old-growth forest sites, but according to the “Atlas dos Remanescentes Florestais da Mata Atlântica” [4] most of deforestation in these sites is illegal.

Some issues must be considered in this work. Both fire data and deforestation data were extracted from annual accumulations of the MapBiomas Project database, with the possibility of overlapping data from one year to the next. That is because the deforestation recorded in one year may have been maintained in the following year if a fragment has not regenerated or converted into another land use and land cover; however, we believe this represents a very small percentage of the total deforestation, if it occurs, as rural land in São Paulo state is quickly destined for other uses after deforestation.

In addition, MapBiomas fire data maps all fire scars visible in satellite images, with moderate to high severity fire, based on differences between pre and post fire NBR index [31], so low intensity and under canopy fires that do not produce scars were not quantified. Thus, we stress that this study can only tell us whether deforestation occurred following moderate to high-intensity fire (sufficiently intense to be detected by MapBiomas). Furthermore, this work evaluated the relationship between deforestation following fire and not the opposite, because field observations and communications with other researchers and environmental technicians indicate that fire is being used to intentionally degrade tropical forests and then, deforest them. As already discussed, fire origins are unknown (probably humans), but some recent data point that they are deliberately ignited to cause degradation and increase tensions for land transformation (especially related to large agriculture farms and agri-companies: [52].

Little attention has been given to fire threats to the Atlantic Forest Biome [17, 22] and its relation with deforestation. Fire events in this Biome may become more common with climate change [53] and they would inhibit forest restoration and conservation [22, 54], especially in São Paulo state that concentrates forest restoration efforts (observatoriodarestauracao.org) and several Atlantic rainforest protected areas. Besides ecological losses, health and socioeconomic issues should be considered as the biome concentrates the largest portion of the Brazilian population and the greatest GDP of the country. Likewise, a National Policy for Management and Control of Fires is urgently needed.

## Conclusion

This study provides the first evaluation of a possible relation between fire and deforestation in the Atlantic Forest biome, southeast Brazil. We expected that deforestation would follow burnings, what we partially observed. Burning positively influenced deforestation in EGF in eight of 19 years studied (2001, 2004, 2005, 2007, 2008 and 2009, 2013 and 2015), while only for three years in the SSF. Burning followed by deforestation corresponded to only 3.2% of the total deforestation, located mainly in the eastern region of the state with the highest density in the EGF. Most of these areas have been converted to commodities and large-scale agriculture, as we expected. This study provides the first indication that, generally, fire is not a driver of deforestation in the southeast Atlantic Forest nowadays. The use of fire for land clearings is not well documented in the Atlantic Forest and makes it difficult to understand if sites would be opportunistically deforested after an accidental fire event or if burnings would be criminally intended to degrade forest sites to be later replaced by another land use.
